# New Clinical Phenotype of the Post-Covid Syndrome: Fibromyalgia and Joint Hypermobility Condition

**DOI:** 10.3390/pathophysiology29010003

**Published:** 2022-01-19

**Authors:** Natalia Gavrilova, Lidiia Soprun, Maria Lukashenko, Varvara Ryabkova, Tamara V. Fedotkina, Leonid P. Churilov, Yehuda Shoenfeld

**Affiliations:** 1 Laboratory of the Mosaic of Autoimmunity, Saint Petersburg State University, 199034 Saint Petersburg, Russia; lidas7@yandex.ru (L.S.); pushisti.legolas@mail.ru (M.L.); varvara-ryabkova@yandex.ru (V.R.); t.v.fedotkina@gmail.com (T.V.F.); elpach@mail.ru (L.P.C.); yehuda.shoenfeld@sheba.health.gov.il (Y.S.); 2 Saint Petersburg Research Institute of Phthisiopulmonology, 191036 Saint Petersburg, Russia; 3 National Medical Research Center named after V. A. Almazova, 197341 Saint Petersburg, Russia; 4 School of Medicine, Ariel University, Ariel 4076414, Israel; 5 Zabludowicz Center for Autoimmune Diseases, Sheba Medical Center, Ramat Gan 5265601, Israel

**Keywords:** fibromyalgia, post-COVID-19 condition, coronavirus infection, autoimmunity, chronic fatigue syndrome, musculoskeletal disorders, pain syndrome, joint hypermobility

## Abstract

Fibromyalgia can be defined as a chronic pain condition, affecting the musculoskeletal system, etiology and pathophysiology of which is sufficiently understudied. Despite the fact that many authors consider this entity to be a manifestation of central sensitization, and not an autoimmune disease, the high prevalence of fibromyalgia in patients with post-COVID-19 conditions requires taking a fresh look at the causes of the disease development. During the patient examination, the authors identified a combination of symptoms that occurs so often, that they can be carefully described as a clinical pattern. These manifestations include young age, female gender, joint hypermobility, the onset of pain after COVID-19, physical traumatization of one particular tendon and the development of the fibromyalgia pain syndrome during the next several weeks. As well as an increase in the titer of antinuclear antibodies and some other systemic inflammation factors. It can be assumed with great caution that local damage to the connective tissue in patients with joint hypermobility, having COVID-19 as a trigger factor can lead to the development of fibromyalgia syndrome. This article presents three clinical cases that illustrated this hypothesis.

## 1. Introduction

According to the 2016 American College of Rheumatology (ACR) criteria, fibromyalgia can be described as a chronic disease with widespread musculoskeletal pain. Its characteristics include spreading of pain in at least four or five body areas, the presence of these symptoms for at least three months, as well as more than 7 points on the Widespread pain index (WPI) and more than 5 points on the symptom severity scale (SSS) or WPI 4–6 and SSS score ≥9 [1]. The true prevalence of fibromyalgia is unknown but can be up to 2–5% of the population in developed countries, creating a prominent socio-economic impact and dramatically lowering the quality of life of the patients [2].

The etiology and pathogenesis of fibromyalgia are still substantially understudied. Many researchers believing, that this disease is not of autoimmune nature and is not characterized by inflammation of any kind in peripheral tissues, but instead is a result of central sensitization and impaired pain processing in the brain [3,4,5,6]. Some researchers have also suggested the involvement of the dorsal root ganglia and in particular the role of ion channel genotypes [7], as well as the possible role of small fiber neuropathy [8,9]. The last statement seems to be especially important due to the fact that fibromyalgia is most often accompanied by such manifestations as chronic fatigue, brain fog, sleep disturbance and autoimmune dysautonomia, which makes it possible to allude its connection with chronic fatigue syndrome and autoimmune/inflammatory syndrome induced by adjuvants (ASIA) syndrome [9,10,11,12]. During the epidemic of a new coronavirus infection, there were several publications, describing fibromyalgia syndrome in patients with the long-COVID-19 condition, that began to be an important problem of modern health care [12,13].

In 2021, the center for the diagnosis and treatment of patients with post-covid syndrome was opened at the clinic of high medical technologies of St. Petersburg State University (Saint-Petersburg, Russia). During the patients’ examination, several clinical patterns of post-covid syndrome were identified, one of which was the fibromyalgia syndrome (FMS). Clinical and laboratory manifestations of this syndrome in the examined patients are so similar, that a single pathogenesis for the development of this disease may be cautiously alluded to. In this paper three typical clinical observations of patients with post-covid FMS are described, which can illustrate this assumption. At the end of the article, a scientific hypothesis is formulated, which requires careful verification and further clinical studies.

## 2. Clinical Case № 1

A 28-year-old woman came to the appointment with severe myalgia and arthralgia, which started after a new coronavirus infection she suffered 6.5 months before. Other complaints included persistent fatigue, brain fog, difficulties in concentration, shortness of breath, and a burning sensation between the shoulder blades. During the COVID-19 infection in this patient, the condition was mildly severe, without the need for mechanical ventilation or stay at the intensive care unit. After being discharged from the hospital, she felt relatively well, having only attention difficulties and hair loss. Three weeks after the hospitalization she decided to start running for 30–40 min every evening. During the workout, she noted tendon pain in the right popliteal fossa. The tendon became swollen and painful and was palpable under the skin like a fibrous cord. She did not get any medical help, hoping for a quick recovery. However, over the next few weeks, she began to notice pain in the ligaments of the right shoulder, in the muscles of the occipital region, then in the muscles of the upper and lower extremities. She had a course of NSAIDs without a pronounced positive effect. At the current moment, 5 months after the onset of the pain syndrome, the patient noted persistent pain in the muscles of the trunk and extremities, in the joint area and periarticular ligaments. Daytime pain constantly had an intensity of 5–6 out of 10 points on the visual analog pain scale and intensifies at night, which causes sleep disturbance.

### 2.1. Examination

During the examination, an active orthostatic test was performed, and postural orthostatic tachycardia (POTS) was detected. There was also a presence of joint hypermobility syndrome (7 points out of 9 on the Beighton scale), which the patient had not noticed before and which did not bother her in any way. Otherwise, the physical examination was normal. Areas of the joints were without edema or redness. The patient had 11 points in the widespread pain index (WPI) and 9 points in the symptom severity scale (SSS). Palpation revealed pain in 12 of 18 tender points, as well as the presence of swollen, sore tendons in these places. Neurological examination within normal ranges.

### 2.2. Instrumental and Laboratory Findings

Electroneuromyography, MRI of the spine and brain, X-ray of the knee and shoulder joints without pathological findings. After a consultation with a rheumatologist, laboratory examinations and a list of autoantibody tests were prescribed, of which only C-reactive protein and antinuclear antibodies were positive. A diagnosis of fibromyalgia was evaluated. Physical rehabilitation and pain management therapy were prescribed.

## 3. Clinical Case № 2

A 45-year-old patient, a female, complained of impaired memory and concentration, as well as severe pain in muscles and joints, after a new coronavirus infection disease that she suffered 5 months ago. Similar to the previous case, the patient also had mild COVID-19 disease and immediately after discharge from the hospital felt moderately well, with the exception of some weakness. Two weeks after being discharged, her dog was injured and could not walk the stairs on its own, and therefore, the patient began to carry it for a walk on her hands. At this moment, the patient began to notice pain in the right knee, which at first did not bother her much. However, the pain syndrome progressed and after 4 weeks a fibromyalgia pattern developed with widespread muscle and joint pain, thickening and tenderness of tendons. In the daytime, the pain had a moderate intensity of 4 points out of 10 on the visual analog scale of pain, but at night it reached 7–8 points out of 10, which made the patient get up and walk around the apartment for a long time in order to reduce the intensity of the pain and try to sleep again.

### 3.1. Examination

During the examination, an active orthostatic test was performed. Postural orthostatic tachycardia was not detected, however, the increase in heart rate between lying and standing position was 27 beats within 3 min, which can be regarded as a borderline result. The presence of hypermobility syndrome (5 points out of 9 on the Beighton scale) was also noted. Areas of the joints were not swollen, without redness and pain during the examination. The patient had 9 points WPI and 6 points SSS. Palpation of the tender points revealed pain in 10 of 18 of them. Otherwise, the physical examination was interpreted as normal. Neurological examination was without pronounced dysfunction.

### 3.2. Instrumental and Laboratory Findings

Electroneuromyography, cervical and brain MRI, X-ray of the knee joints were without pathology. A consultation with a rheumatologist was performed and laboratory analysis and autoantibodies tests were prescribed, of which only antinuclear antibodies titers were high, as well as an increase in ESR up to 22 mm/h. A slight increase in rheumatoid factor titer and borderline lymphocytosis were also noted. A patient was diagnosed with fibromyalgia and symptomatic treatment was prescribed.

## 4. Clinical Case № 3

A 37-year-old female patient came to the appointment with severe fatigue, memory impairment, hair loss, shortness of breath and severe pain in the shins and feet on both sides. About eight months ago, she had a new coronavirus infection, after which the described above symptoms were raised. The COVID-19 infection itself was mild and she was not hospitalized. Immediately after recovery, severe weakness and shortness of breath emerged. Two months later, to improve her health condition, she started to swim for one hour three times a week. After several swim sessions, she began to notice pain in the right, and then in the left ankle. Later she noticed that pain spread up her legs and reached the knees.

### 4.1. Examination

A postural orthostatic tachycardia was revealed. The presence of joint hypermobility (6 points out of 9 on the Beighton scale) was noted. Otherwise, the physical examination was normal. Areas of the joints were not swollen, without redness and pain. Despite the fact that the patient did not mention muscle pain in the body and upper extremities, palpation revealed pain in 12 out of 18 tender points with swollen, painful tendons. The patient had 11 points WPI and 8 points SSS. The neurological examination also revealed a distortion of pain sensitivity with tingling and burning in the area of the feet and ankle joints on both sides, which also appeared after the new coronavirus infection.

Instrumental and laboratory findings. Electroneuromyography, brain MRI, X-ray of the knee and ankle joints were without pathology. Metabolic diseases as a cause of polyneuropathy were excluded. A panel of laboratory tests with antibody titers evaluation was prescribed. Higher than normal levels of antinuclear antibodies was evaluated, as well as lymphocytosis and an increase in C-reactive protein up to 17 mg/L. Fibromyalgia was diagnosed and symptomatic treatment was prescribed. Considering the signs of depression, the patient was referred to a psychiatrist for the diagnosis establishment and therapy prescription. 

### 4.2. Discussion

The presented three clinical cases illustrate a pattern that can be distinguished in post-covid syndrome. These are mainly female patients, of a young age (20–45 years old), with the presence of joint hypermobility syndrome. The new coronavirus infection had a mild to moderate severity. The authors do not have observations of severe cases with prolonged hospitalization. After the infection, for several weeks, the patients have felt normal, but not completely healthy. The most common complaints included weakness, dizziness, memory impairment, hair loss, shortness of breath, and burning sensations in the chest. The described complaints made patients increase their physical activity. After a few weeks, the patients began to notice pain in one tendon, or simply “in the joint” or “in the limb.” Most often, they didn’t have any medical care or take non-steroidal anti-inflammatory drugs without a pronounced positive effect. A few more weeks later, the classical pattern of fibromyalgia develops, confirmed by a rheumatologist.

A number of features that characterize these patients should be mentioned. First, the patients often had less severe pain sensations than typical idiopathic fibromyalgia. The pain syndrome is often quite tolerable and can be as low as 3–4 points on the VAS, in some cases, without requiring the pain management medications. Second, the manifestations are usually associated with autonomic disorders (POTS) and polyneuropathy, as in cases 1 (burning sensation between the shoulder blades) and 3 (burning feeling in the legs and chest). Third, in such patients, anxiety and depression are often observed, however, there is an impression that these disorders are secondary to pain syndrome, sleep deprivation and stress from not understanding the causes of their disease. An exception is case 3, where the depressive background accompanied the patient for several years and, possibly, is the reason that her complaints were the least specific and difficult to interpret among the described clinical cases. Fourth, it seems important that the titer of antinuclear antibodies, C-reactive protein and ESR were increased, while other laboratory signs of autoimmune processes are usually negative. Fifth, joint hypermobility syndrome is having a high overlap—up to 80%—with fibromyalgia and chronic fatigue syndrome [14,15,16,17,18]. Eccles et al. in their studies also provided evidence both for the overlap between hypermobile Ehlers–Danlos syndrome and FM, as well as between FM and CFS (up to 90%), which may allude that, that disease may be a part of one spectrum [18,19]. In addition, in patients with the Marfanoid phenotype, a high level of TGF beta1-beta2 can be expected, which worsens the clinical course and prognosis of COVID-19 [20,21,22]. 

There are a growing number of studies that are declaring the link between post-COVID-19 condition and FM syndrome. Dotan and Shoenfeld described autonomic dysfunction, pain syndromes and elevated autoantibodies titers in sera of the novel coronavirus infection convalescents. Autoantibodies to β2-adrenoceptor, α1-adrenoceptor, and angiotensin II receptor type 1 receptor are of particular interest [23]. Ursini et published important data, where up to 30% of patients with the post-COVID syndrome may satisfy the criteria for FM. Notably, obesity and male gender were described as the strongest risk factors for FM complications [24]. Finally, more than 900 patients with FM were examined for the effect of coronavirus infection on their condition. Most often, patients noted an increase in precisely those symptoms that (in addition to pain) are most typical of FM, such as fatigue, sleep disturbances and muscle stiffness. Therefore, the core symptoms of idiopathic FM worsened after the COVD-19 infection, which may be a result of physical and mental stress, or the neurotoxicity of the virus somehow affects the autonomic regulation in patients with FM [25].

Antinuclear antibodies for many years were considered to be key markers in the evaluation of rheumatic diseases, most prominently systemic lupus erythematosus (SLE) [26]. As early as in the 1980s, ANA autoantibodies were evaluated in idiopathic FM patients, which suggested a link between FM and connective tissue diseases [27]. On the other hand, other studies didn’t prove this data [28,29], therefore, the question needs further investigation. Hafiz et al. provided promising results, where chronic fatigue was common in ANA(+) individuals lacking sufficient criteria for the diagnosis of a rheumatic disease. Additionally, in their study, fatigue correlated with fibromyalgia-related symptoms, and at the same time was not associated with systemic inflammation [30]. Recently, studies have shown an increase in antinuclear antibodies titers in patients with novel coronavirus infection, which requires further research to assess their prognostic role in the development of an autoimmune response in this condition. [31].

Thus, it is possible to propose with great caution a clinical case definition of fibromyalgia syndrome with hypermobility developing after a COVID-19 infection. It is known that the overlap of joint hypermobility syndrome and fibromyalgia reaches 80%, however, to our best knowledge, no explanation of this phenomenon has appeared. The development of pain syndrome after exercise and the onset of fibromyalgia from one tendon may allude to a traumatized connective tissue in such patients with joint hypermobility. COVID-19 as a trigger factor can lead to immunologic reactions to proteins of the connective tissue and the development of fibromyalgia syndrome.

## Data Availability

All data generated or analyzed during this study are included in this published article.
